# Recent Findings in Azaphilone Pigments

**DOI:** 10.3390/jof7070541

**Published:** 2021-07-07

**Authors:** Lúcia P. S. Pimenta, Dhionne C. Gomes, Patrícia G. Cardoso, Jacqueline A. Takahashi

**Affiliations:** 1 Department of Chemistry, Universidade Federal de Minas Gerais (UFMG), Av. Antonio Carlos, 6627, Belo Horizonte CEP 31270-901, MG, Brazil; lpimenta@qui.ufmg.br; 2 Department of Food Science, Universidade Federal de Minas Gerais (UFMG), Av. Antonio Carlos, 6627, Belo Horizonte CEP 31270-901, MG, Brazil; dhionne@gmail.com; 3 Department of Biology, Universidade Federal de Lavras, Av. Dr. Sylvio Menicucci, 1001, Lavras CEP 37200-900, MG, Brazil; patricia@ufla.br

**Keywords:** natural pigments, filamentous fungi, azaphilones, production, biotechnological tools, non-mycotoxigenic strains, regulatory issues

## Abstract

Filamentous fungi are known to biosynthesize an extraordinary range of azaphilones pigments with structural diversity and advantages over vegetal-derived colored natural products such agile and simple cultivation in the lab, acceptance of low-cost substrates, speed yield improvement, and ease of downstream processing. Modern genetic engineering allows industrial production, providing pigments with higher thermostability, water-solubility, and promising bioactivities combined with ecological functions. This review, covering the literature from 2020 onwards, focuses on the state-of-the-art of azaphilone dyes, the global market scenario, new compounds isolated in the period with respective biological activities, and biosynthetic pathways. Furthermore, we discussed the innovations of azaphilone cultivation and extraction techniques, as well as in yield improvement and scale-up. Potential applications in the food, cosmetic, pharmaceutical, and textile industries were also explored.

## 1. Introduction

Color has been used by mankind since the Neolithic period and has been associated to different people such as purple to the Phoenicians, yellow (annatto) to the Mayans, and to different purposes as henna pigments for body and hair coloring in India. In human history, color gained a powerful status in many daily experiences and key decisions. Some studies show, for example, that preference for blues and reds (at the expense of yellowish and greenish hues) influenced auction prices, as reported for Mark Rothko’s rectangular paintings [1].

Color is also naturally associated with chemosensory perceptions regarding flavor, quality and freshness, highly interfering in product choice [2]. In this way, consumers expect some foods to have specific colors. However, variation and heterogeneousness of natural color in foods initiated the process of adding pigments to maintain color uniformity while granting high coloring power, as well as stability in aqueous phase and in different pH [3].

Vegetal-derived natural products are source of pigments very important to the food industry. However, the production is limited by yield issues since the gross amounts of vegetal pigments recovered, even from improved cultivars is not sufficiently competitive to fulfill modern industrial demand. Yield improvement is surely the major problem which have been addressed by developing and breeding modified cultivars and new large-scale processes were developed to the production of natural pigments [4]. Insect-derived coloring compounds such as carmine have been introduced in the market, but despite their natural origin, they are not accepted by many countries’ regulatory agencies due to ethical issues. In addition, vegetarian, vegan and kosher diet adepts do not accept colorants of animal origin [5].

To date, industry still has not overcome the low availability of natural pigments. A successful alternative was found in the synthesis of coloring agents structurally identical to the natural ones to produce compounds like beta carotene, riboflavin and cantaxanthin xanthophylls (yellow, orange and red palette) [6]. However, the synthesis of natural pigments did not bring enough economic competitiveness and there are issues related to the classification (natural or synthetic) of compounds naturally occurring produced by synthetic means. 

With the growing demand for industrialized food, the high per-unit cost of natural colorants boosting the cost of final food products, without the benefit of significant color content, led industries to adopt synthetic substitutes, which feature vast color spectrum, and colorfastness. Azo dyes are some of the most utilized synthetic compounds in this area, offering reproducible stable color. They can be easily synthesized by diazotation of aromatic amines and became the first-choice colorants in food industry for decades [7]. However, azo compounds have been associated with several diseases, including cancer [8]. Moreover, although controversial, meta-analysis studies found evidence on the relationship between intake of artificial food coloring agents with allergic response and behavioral problems such as hyperactivity in children [9]. These facts led regulatory agencies to ban some synthetic colorants and, consequently, food industry is facing the challenge of developing novel formulations containing natural food coloring agents to provide or complement the color palette of foods.

The replacement of artificially colored products by natural ones is also demanded by a new generation of green-minded consumers seeking for “clean label” and safe ingredients. The boom of groups opting for environmentally friendly consumption and healthy lifestyles led to a big change in food consumer behavior, especially by individuals from the so-called Generation Z (Gen-Z). This group was pointed to account for about 40% of all consumers, the largest consumer market share in 2020 [10].

In this scenario, fungi are highly quoted as alternative sources of naturally derived, healthy, safe, stable and low-cost pigments for food industry applications [11]. Fungal bio-pigments have the advantage of being produced using inexpensive sources of carbon and nitrogen, that can even be obtained from food by-products or from agro-industrial residues [12]. One of the most promising classes of fungal pigments in research as industrial pigments are azaphilones, compounds that stand out for their yellow, orange and red colors [13]. This class of fungal secondary metabolites encompasses a large number of compounds of polyketide origin, containing a pyrone-quinone core, a chiral quaternary center and hydroxyl groups as substituents. Orange-colored azaphilones usually possess a heterocycle containing a pyranyl oxygen that is susceptible to aminophilic reactions where the pyran oxygen atom is exchanged for a nitrogen atom derived from peptides, nucleic acids, proteins and others [14]. This exchange alters the absorption of the pigment that goes from orange to red, frequently also altering the biological properties.

Azaphilones research is extremely important and literature reporting new azaphilone derivatives described in the last decades, different fungi sources, and a wide scope of biological activities is comprehensive. However, many issues on industrial scaleup of wet bench fermentative conditions, optimized production, efficient extraction protocols to maximize industrial production and certification of generally recognized as safe (GRAS) strains are areas that still demand research and technological development. An expressive number of works have been addressing the challenge to find a safe, low cost azaphilone source to fit the contemporary demand for edible natural pigments that meet regulatory guidelines. The readiness of fungi-derived red colorants for use in food industry was discussed on an interesting paper by Dufossè [15], while production of yellow pigments by *Monascus* sp. was addressed by Yang et al. [16].

This review, covering literature from January 2020 to April 2021, focuses on the state-of-the-art of azaphilone research, comprising market scenario, fungi sources reported in the period, main cultivation, extraction, and purification techniques, chemistry, scope of biological activity, and potential applications in the food industry. Strategies for yield improvement and scale up, associated with market possibilities for cosmetic, pharmaceutical, and textile industries among other applications will also be discussed. 

## 2. Global Market Size for Yellow, Orange and Red Colored Pigments

The economic crisis imposed by COVID-19 pandemic in 2020 profoundly influenced human life in several ways. Industrial sector suffered huge losses and long-term effects are expected in all sectors that had to adapt marketing policies to minimize economic breakdown [17]. Nevertheless, the market of additives for food industry may achieve 43.3 billion USD by 2021 [18] with the food colorant market alone being responsible for 3.75 billion USD [19]. The contribution of global food pigment (comprising carotenoids, caramel, curcumin and spirulina among others) market is projected to reach 1271.4 million USD by 2025 [20].

Although the good scenario and ascending numbers, prohibition or scrutiny of some artificial red colorant compounds and legal restrictions to their use in the food industry either by the Food and Drug Administration in the USA or by the European Food Safety Agency (EFSA) have been affecting the availability of food pigments and the color palette. A major issue related to synthetic compounds regards the azo-aromatic group present in the chemical structure of many red and yellow synthetic colorants. In the same way, allergy and other allegations have also been affecting the employment of yellow pigments such as tartrazine in foodstuff [21]. This becomes a problem, as the range of color yellow-red is essential in the food industry. Red, yellow, and orange, along with “clear” and white colors were associated with refreshing foods and beverages [22]. Even though the role of color in the market “is still in infancy”, high color saturation captures consumer attention. For example, red color is protagonist in avoidance or approach motivations related to fresh fruits preference, as red color is associated with fruit ripening [23]. Color perception, together with visible fat and origin were reported as the main intrinsic attributes that drive choice of pork products by consumers from some emerging markets [24].

**Figure 1:**
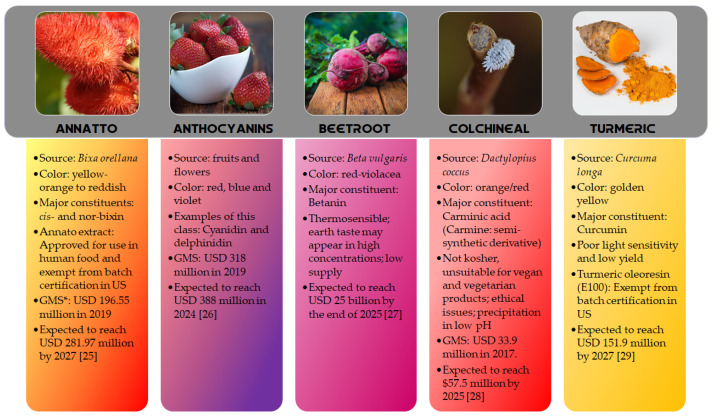
Source, color, constitution and global market size (GMS) data for some vegetal-originated colored compounds [25,26,27,28,29].

Innumerous compounds biosynthesized by plants have yellow, orange and red natural color, as determined by structural features. Some of them meet the requirements for using in food products such as anthocyanins (cyanidin), carotenoids (bixin), indole-derived glycosides (betanin), anthraquinones (carminic acid) and polyphenols (curcumin). Some information and market size for these pigments can be found in Figure 1. 

## 3. Chemistry, Biological Activities and Biosynthetic Pathways of Recently (2020–2021) Reported Azaphilones

### 3.1. New Azaphilone Compounds 

Two complementary reviews cover a good part of literature about azaphilones from 1932 to 2019. Gao et al. [30] reviewed literature from end of 1932 to September 2012, reporting data on 373 azaphilones of 18 categories and Chen et al. [31] published data on the chemistry and biology of azaphilones, covering 252 compounds predominantly originated from 32 genera of fungi reported between October 2012 to December 2019 [31]. Naturally-derived azaphilones reported by Chen et al. [31] were classified in 13 types: nitrogenated, citrinins, austdiols, deflectins, bulgariolactones, spiro-azaphilones, O-substituted, lactone, hydrogenated, chaetovirins, pulvilloric acid, sclerotiorins, and cohaerins. Azaphilone pigments of atrorosin class produced by *Talaromyces atroroseus* were reviewed by Isbrandt et al. [32], and Morales-Oyervides et al. [11] reviewed natural colorants produced by fungi from *Talaromyces*/*Penicillium* genus [11].

**Figure 2:**
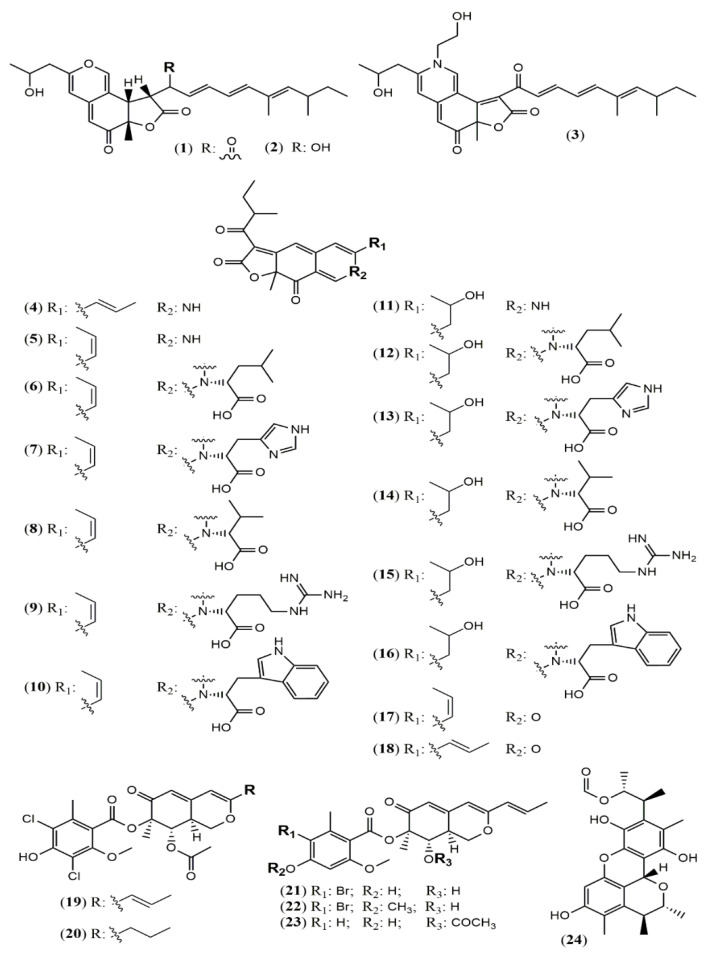
Chemical structures of *Aspergillus* azaphilones **1–2**: sassafrin E-F; **3**: sassafrinamine A; **4**: *trans*-cavernamine; **5**: *cis*-cavernamine; **6–10**: Leu, His, Val, Arg, Trp-cavernamine derivatives; **11**: hydroxy-cavernamines; **12–16**: Leu, His, Val, Arg and Trp-hydroxy-cavernamines.; **17**: *cis*-cavernine; **18**: *trans*-cavernine; **19–20**: falconensins O and P; **21–23**: falconensins Q, R, and S; **24**: penicitrinol Q [33,34,35,36].

**Figure 3:**
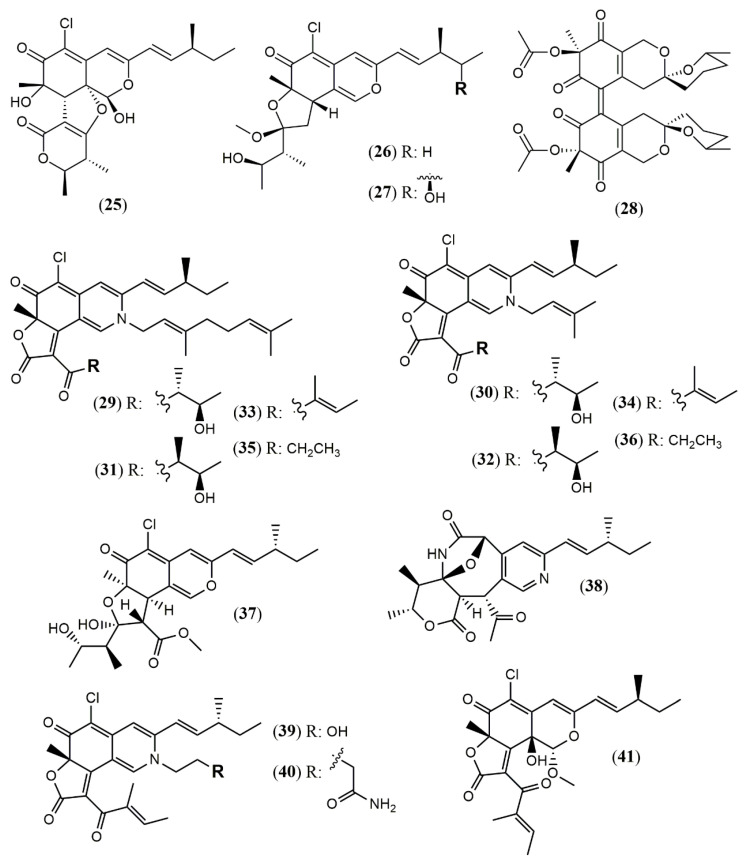
Chemical structures of *Chaetomium* azaphilones: **25**: Chaephilone C (1R,7S,8R,8aR,9E,11S,4′R,5′R); **26**: chaephilone D; **27**: chaephilone C*; **28**: cochliodone J; **29**: N-(3,7-Dimethyl-2,6-octadienyl)-2-aza-2-deoxychaetoviridin A; **30**: 4′-epi-N-(3,7-Dimethyl-2,6-octadienyl)-2-aza-2-deoxychaetoviridin A; **31**: N-(3-Methyl-2-butenyl)-2-aza-2-deoxychaetoviridin A, **32**: 4′-epi-N-(3-Methyl-2-butenyl)-2-aza-2-deoxychaetoviridin A; **33**: N-(3,7-Dimethyl-2,6-octadienyl)-2-aza-2-deoxychaetoviridin E; **34**: N-(3-Methyl-2-butenyl)-2-aza-2-deoxychaetoviridin E; **35:** 4′,5′-dinor-5′-Deoxy-N-(3,7-dimethyl-2,6-octadienyl)-2-aza-2- deoxychaetoviridin A; **36**: 4′,5′-dinor-5′-Deoxy-N-(3-methyl-2-butenyl)-2-aza-2-deoxy- chaetoviridin A; **37**: seco-chaetomugilin; **38**: chaetolactam A; **39**: 11-epi-chaetomugilide B; **40**: chaetomugilide D; **41**: globusumone [37,38,39,40,41,42,43,44,45].

**Figure 4:**
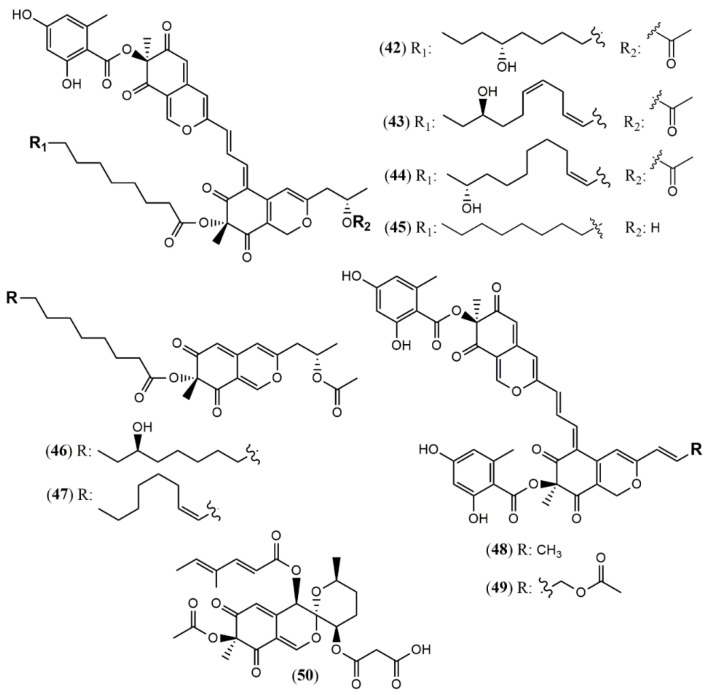
Chemical structures of *Hypoxylon* azaphilones: **42–45**: hybridorubrins A–D; **46–47**: fragirubrins F and G; **48–49**: rutilins C–D; **50**: 3′-malonyl-daldinin F [47,48].

**Figure 5:**
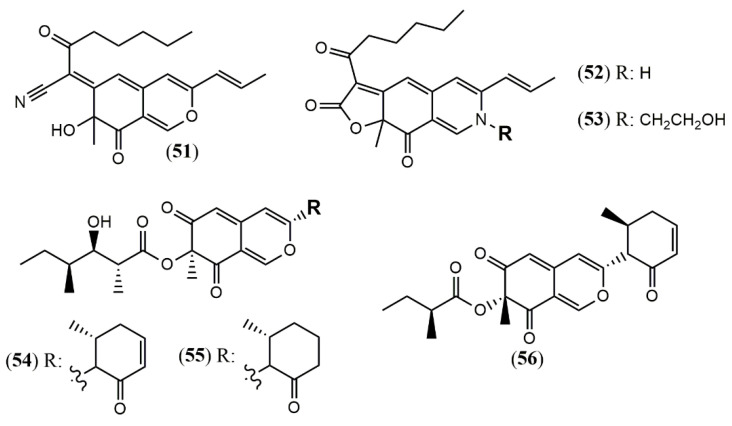
Chemical structures of *Monascus* and *Muyocopron* azaphilones: **51**: monapilonitrile; **52**: monapilosine; **53**: N-ethanolic monapilosine; and *Muyocopron* azaphilones: **54–55**: muyocoprones A and B; **56**: lijiquinone 1 [50,51].

**Figure 6:**
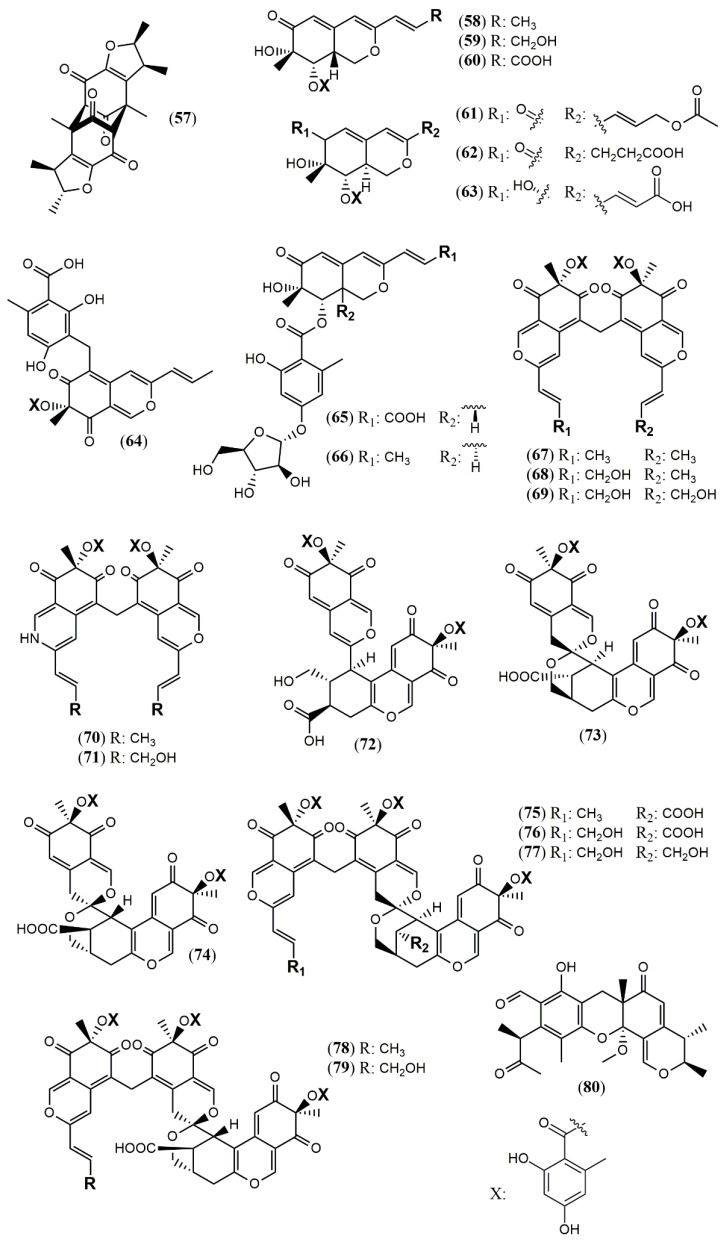
Chemical structures of *Penicillium azaphilones*: **57**: penicitrinone G; **58–64**: Dangelones A–G; 65–66: dangelosides A and B; **67–74**: didangelones A–H; **75–79**: tridangelones A–E; **80**: penctrimertone [52,53,54].

**Figure 7:**
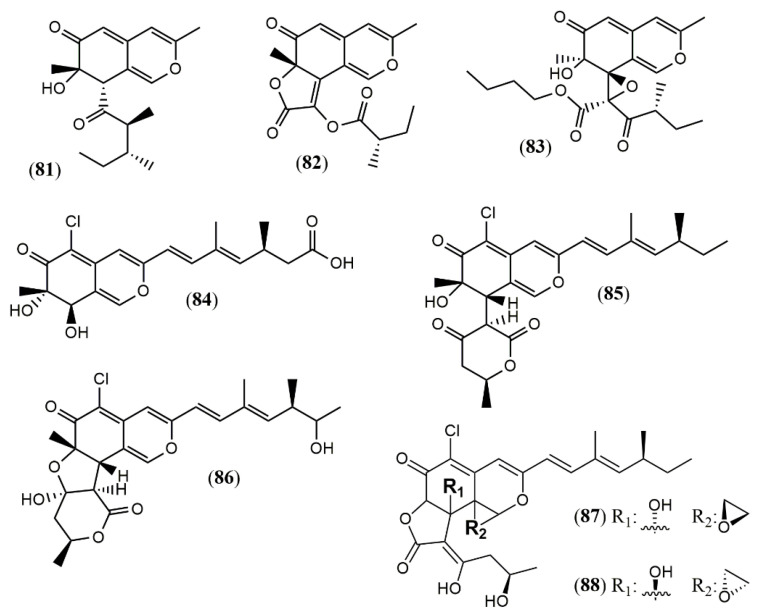
Chemical structures of azaphilones from *Phomopsis*: **81–83**: phomopsones A–C; **84–88**: tersaphilones A–E [55,56].

**Figure 8:**
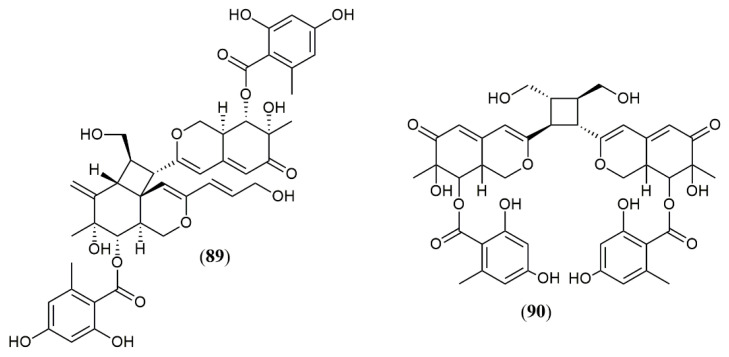
Chemical structures of azaphilones from *Pleosporales*: **89–90**: pleosporales A and B [57].

**Figure 9:**
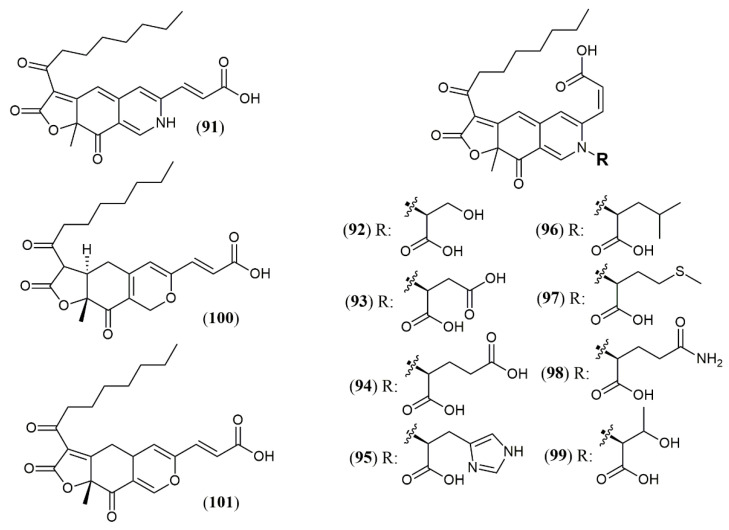
Chemical structures of *Talaromyces* azaphilones: **91**: trans-PP-O; **92–99**: atrosins S (Ser),D (Asp), E (Glu), H (His), L (Leu), M (Met), Q (Gln), and T (Trp); **100–101**: talaralbols A and B [32,58].

In this section, it is presented a summary of the new compounds reported after December 2019 classified according to the fungal genera source. Despite the great number of 100 new compounds reported from January 2020 to March 2021, the azaphilones were isolated only from nine fungal genera (*Aspergillus*, *Chaetomium*, *Hypoxylon*, *Monascus*, *Muycopron*, *Penicillium*, *Phomopsis*, *Pleosporales,* and *Talaromyces*). The genus *Phomopsis* was not cited in the latest review and now appeared as fungal endophytic sources of chlorinated azaphilone pigments. At this time, it will be presented the new compounds isolated from each genus (Figures 2–9) displayed according to the species (Table 1).

#### 3.1.1. Azaphilones from *Aspergillus* Genus

*Aspergillus* genus is one of the three largest genera where azaphilones can be found. Recently, 23 azaphilones (**1–23**) were isolated from three species (Figure 2 and Table 1). Sassafrin E (**1**), sassafrin F (**2**), and sassafrinamine A (**3**) were isolated from the filamentous fungus *Aspergillus neoglaber* 3020 [33]. Two pigments (Sassafrin E (**1**) and Sassafrin F (**2**)) were yellow and display the azaphilone core fused to the same angular lactone ring with different substituents. The third pigment (sassafrinamine A) (**3**) is purple and displays a nitrogen into the isochromene system substituted with ethyl-1-ol group (Figure 2). The fungus *Aspergillus cavenicola* afforded the nitrogenated azaphilones *trans*-cavernamine (**4**), *cis*-cavernamine (**5**), amino acid derivatives of *cis-*cavernamines (**6–10**), hydroxy-cavernamine (**11**), amino acid derivatives of hydroxy-cavernamines (**12–16**), and two oxygenated derivatives *cis* and *trans*-cavernines (**17–18**) [34]. The marine-derived fungus *Aspergillus falconensis* yielded five mitorubrins derivative azaphilones with different benzoyl moieties: two new chlorinated azaphilones, falconensins O and P (**19** and **20**) when the fungus was cultivated in a solid rice medium containing 3.5% NaCl and three additional new azaphilone derivatives (**21–23**) when NaCl was replaced by 3.5% NaBr [35]. From the endophytic *Aspergillus terreus* of *Pinellia ternate,* the undescribed dimer of citrinin penicitrinol Q (**24**) was isolated displaying accentuated Gram-positive antibacterial activity [36].

#### 3.1.2. Azaphilones from *Chaetomium* Genus

*Chaetomium* is a large genus presenting more than 300 species worldwide. *Chaetomium globosum* represents one of the most studied species and is known as a rich source of azaphilones. Since the last two years, this species has still been contributing with new metabolites. The arthropod-associated endophytic fungus *C. globosum* TW1–1 was investigated considering whether the presence of 1-methyl-l-tryptophan into the growth medium would activate a biosynthetic pathway to produce novel alkaloids [37]. However, instead of nitrogenated metabolites, the authors isolated and identified two chlorinated azaphilones, chaephilone C and D (**25–26**) with anti-inflammatory activity. Their stereostructures were unequivocally confirmed by X-ray analyses. Nevertheless, chaephilone C was also previously reported from the deep sea-derived fungus *Chaetomium* sp. NA-S01-R1 with the same planar structure of **25** but with different stereochemistry, suggesting that its structure should be revised [38]. Two months after the report of chaephilone C (**25**), a new chlorinated azaphilone from *C. globosum*, endophytic of *Polygonatum sibiricum*, was reported and also called chaephilone C (**27**) [39]. However, this latter compound displayed a chemical structure similar to (**26**), but completely different from the former (**25**).

From the wild-type strain *C. globosum*, a new dimeric azaphilone called cochliodone J (**28**) was identified in the same medium which cochliodone A had been isolated before [40]. The deep-sea *C. globosum* MP4-S01–7 provided eight new structurally correlated nitrogenated azaphilones **29–36** (Figure 3 and Table 1) [41]. The azaphilone core is the same in all compounds with differences only in the lactone acyl substituents and the N-alquil groups. Seco-chaetomugilin (**37**) was isolated for the first time from the ethyl acetate extract of *Chaetomium cupreum* in a bio-guided fractionation for activities against human breast adenocarcinoma cell lines [42]. Although the authors named the compound isolated as seco-chaetomugilin, it presented the same structure of seco-chaetomugilin D, previously isolated from *C. globosum* [43]. A screening by LC-MS/MS-GNPS data base of a strain of an endophytic plant fungus *Chaetomium* sp. g1 resulted in the isolation of chaetolactam A (**38**), a unique 9-oxa-7-azabicyclo[4.2.1]octan-8-onering system with two new compounds chaetoviridins derivatives, 11-epi-chaetomugilide B (**39**), and chaetomugilide D (**40**) [44]. Another plant endophytic fungus *C. globosum* isolated from the desert Asteraceae species, *Artemisia desterorum,* yielded globosumone (**41**), a new stereoisomer of the known chaetoviridin E [45].

#### 3.1.3. Azaphilones from *Hypoxylon* Genus

Four unprecedented bisazaphilones hybridorubrins A–D (**42–45**) were isolated together with two new mitorubrin-type azaphilones, fragirubrins F–G (**46–47**) [46] from *Hypoxylon fragiforme*. The main differences among them are the acyl substituents in the lenormandin/fragirubrin-type moiety. In this study, the authors determined the azaphilones stereochemistry by electronic circular dichroism (ECD) spectroscopy in a comparative study between isolated and synthetic compounds. The acquired data suggest that the previous stereochemistry reported for rutilins C (**48**), D (**49**) and the mitorubrins [47] must be revised to be (S)-configured at C-8 and C-8a (Figure 4). Another species *Hypoxylon fuscum* complex yielded a new daldinin F derivative possessing a 3′-malonyl group (**50**) [48].

#### 3.1.4. Azaphilones from *Monascus* Genus

*Monascus pilosus* BCRC 38072, a citrinin-free strain, was able to produce several azaphilone pigments including three new *Monascus* red pigments without citrinin presence: monapilonitrile A (**51**), monapilosine (**52**), and *N*-ethanolic monapilosine (**53**) [49] (Figure 5). Metabolites (**52**) and (**53**) are nitrogenated azaphilones lacking or bearing the N-hydroxyethyl group, respectively.

#### 3.1.5. Azaphilones from *Muyocopron* Genus

The chemical investigation of the endophyte *Muyocopron laterale* ECN279 isolated from a health leaf of *Conavalia lineata* led to the isolation of the two new azaphilones muyocopronones A and B (**54–55**) [50]. An endophyte fungus F53 from the traditional Chinese medicine plant *Taxus yunnanensis* had its genome sequenced and mined, and the multi-locus phylogeny of F53 allowed its placement within the genus *Muyocopron* with its closest relative being *Muyocopron atromaculans* (MUCL 34983) [51]. Moreover, a new azaphilone lijiquinone 1 (**56**) with activities against human myeloma cells and the yeast *Candida albicans* and *Cryptococcus albidus* was isolated from its ethyl acetate extract (Figure 5).

#### 3.1.6. Azaphilones from *Penicillium* Genus

The *Penicillium* genus produces a great number of azaphilone metabolites [31]. *Penicillium citrinum* WK-P9 was isolated as an associated fungus from the sponge *Suberea* sp., displaying antibacterial activity. The bio-guided chemical investigation of its ethyl acetate extract led to the isolation of a new citrinin derivative called penicitrinone G (**57**) [52]. Genome mining, epigenetic regulation, optimization of culture conditions, and one-strain-many-compounds (OSMAC) were investigated as a possible way to prioritize the production of other polyketide metabolites different than the rubratoxins in *Penicillium dangeardii* [53]. Only the metabolic shunting strategy, based on the deletion of the key gene *rbtJ* encoding PKS for rubratoxins biosynthesis, and the optimization of culture conditions successfully led to the production of 35 azaphilones, from which 23 were new ones. They were identified as nine monomers named dangelones A–G (**58–64**), dangeloside A–B (**65–66**), eight dimers, didangelones A–G (**67–74**), and five trimers, tridangelones A–E (**75–79**) [53] (Figure 6). Dangelones A–G (**58–64**) have the same planar structure and the distinctions among them lay on the side chains at C-3. The differences at C-3 side chain are also present in the dimers. Still regarding *Penicillium* endophytic fungi, a strain of *Penicillium* sp. T2–11 isolated from the rhizomes of the underground portion of *Gastrodia elata* produced a citrinin dimer, named penctrimertone (**80**) [54].

#### 3.1.7. Azaphilones from *Phomopsis* Genus

Culture of the endophyte fungus *Phomopsis* sp. CGMCC No.5416 yielded the three azaphilones phomopsones A–C (**81–83**), presenting anti-HIV and cytotoxic activity [55]. From the deep-sea-derived fungus *Phomopsis tersa* FS441, five chlorinated azaphilones named tersaphilones A–E (**84–88**) presenting unique structures were isolated [56] (Figure 7).

#### 3.1.8. Azaphilones from *Pleosporales* Genus

The marine-derived fungus *Pleosporales* sp. CF09–1 produced the uncommon bisazaphilones dipleosporalones A and B (**89–90**) (Figure 8) [57]. These compounds own a 6/4/6 ring system that might come from a [2 + 2] cycloaddition reaction between two pinophilin B-type monomers and represents the first example of this coupling.

#### 3.1.9. Azaphilones from *Talaromyces* Genus

Most strains previously referred to as *Penicillium* sp. are now classified in the *Talaromyces* species, and some of them have been found to produce yellow and red azaphilone pigments. Two new pigments from *T. atroroseus* were described. The first belongs to the series of known *Monascus* orange azaphilone PP-O pigments, and it was unequivocally elucidated as the isomer *trans*-PP-O (**91**) [32] (Figure 9). The second was the unique azaphilone atrosin S, which presented the incorporation of a serine moiety into the isochromene/isoquinoline system. The fungus cultivation in medium enriched with a specific amino acid as sole source of nitrogen could allow seven atrorosin derivatives (atrorosin D, E, H, L, M, Q, and T, depending on the amino acid incorporated) (**92–99**), which were identified by dereplication using HPLC-DAD-MS/HRMS analysis [32]. From the fungus *Talaromyces albobiverticillius* associated with the isopod *Armadillidium vulgare,* two interesting azaphilone pigments talaralbols A and B (**100–101**) was reported [58]. However, talaralbol B presents the same planar structure of trans-PP-O, early described in *T. atroroseus* [32], in which the C-9 stereochemistry was not reported.

### 3.2. Biological Activities of Azaphilones

Azaphilones, besides being good compounds to replace synthetic pigments, aggregate valuable pharmacological properties. The wide broad range of biological activities that has been reported for azaphilones such as cytotoxic, anti-inflammatory, antimicrobial, antitumoral, antiviral and antioxidant is exemplified in Table 1. 

Concerning the activities regarded to the new 101 azaphilones reported, the cytotoxic and antitumor potential are the most evaluated. Remarkably, compounds (**29**), (**30**), and (**33**) showed the most effective anti-gastric cancer activities (MGC803 and AGS cell lines) with IC_50_ values less than 1 μM, being more active than the positive control paclitaxel (3.8 μM) [41]. Additionally, (**29**) and (**30**) induced apoptosis in a concentration-dependent manner and (**30**) inhibited cell cycle progression. The authors also claim that 3,7-dimethyl-2,6-octadienyl group attached to N-2 contributed to the potent cytotoxic activities against MGC803 and AGS gastric cancer cell lines what can induce new investigations with semi-synthetic azaphilone derivatives possessing this group [11]. The azaphilones (**39**) and (**40**) showed moderate activity against leukemia HL-60 and human breast cancer. However, (**39**) exhibited potent apoptosis induction activity by mediating caspase-3 activation and PARP degradation at 3 μM in leukemic cells HL-60 [44]. Another interesting result was the potent cytotoxic activity showed by the dimeric azaphilones (**89**) and (**90**) against five different human cell lines. (**89**) showed more potent cytotoxicity against MGC-803 than cisplatin and possessed a unique 6/4/6 ring system suggesting the new ring may play an important role in cytotoxicity [57].

A great number of azaphilones present anti-inflammatory activity [35,37,38,42,49,50,52,53,58,59]. The compounds (**21**), (**51**), (**52**) and (**100**) exhibited anti-inflammatory activities due to potent anti-NO production activity, with IC_50_ values of 11.9, 2.6, 12.5, and 10.0 μM, respectively, compared to the known iNOS inhibitor quercetin (34.6 ± 1.4 μM) on lipopolysaccharide (LPS) -induced nitric oxide (NO) production [35,46,57]. The antimicrobial activity of azaphilones also must be highlighted. Two dimeric azaphilones, penicitrinol Q (**24**) and penctrimertone (**80**), showed both excellent inhibitory activities against *B. subtilis* with MIC of 6.2 and 4.0 µg/mL, respectively. Moreover, (**24**) also presented inhibitory activity against bacteria *Staphylococcus aureus* (4.3 µg/mL) and *Pseudomonas aeruginosa* (11.2 µg/mL), and the yeast *C. albicans* (4.0 µg/mL) [36].

In vitro antiviral activity against HIV-1 was detected for phomopsones B and C (**82–83**) (7.6 and 0.5 μM, respectively [52]). Research in antiviral potential of azaphilones may be strengthened as they have been focused as possible drug leads for the development of effective antiviral agents against SARS-CoV-2 [60,61]. This worldwide impact-generated virus draws attention to the difficulty in developing new non-toxic antiviral drugs, as viruses use cell host metabolism for replication. This is corroborated by previous reports of antiviral activity of azaphilone metabolites, such as chermisinone B, isolated from the endophytic fungus *Nigrospora* sp. YE3033, and active against A/Puerto Rico/8/34 (H1N1) in CPE assay (IC_50_ 0.80 μg/mL) with low cellular toxicity on MDCK cells (CC_50_ 184.75 μg/mL) [62]. In vitro HIV-1 replication inhibitory effects in C8166 cells were demonstrated for Helotialins A and B (EC_50_ 8.01 and 27.9 nM, respectively) [63]. In 2019, comazaphilone D was reported as a non-competitive inhibitor of neuraminidase from recombinant rvH1N1 (IC_50_ 30.9 µM) while rubiginosin A was active against H5N1 (IC_50_ 29.9 µM) [64]. The previous knowledge of the antiviral potential of azaphilone derivatives is an advantageous background for the development of new drugs to inhibit SARS-CoV-2.

**Table 1:** Azaphilones fungal sources and reported biological activities.

Name (No).	Producing Strains	Activity
*Aspergillus*		
Sassafrin E-F (**1–2**)	*A. neogabler* IBT3020 [33]	Data not reported
Sassafrinamine A (**3**)		
*Trans*-cavernamine(**4**)	*A. cavernicola* [34]	Data not reported
*Cis*-cavernamine (**5**)		
*Cis*-cavernamines-Leu, His, Val, Arg, Trp (**6–10**)		
Hydroxy-cavernamine (**11**)		
Hydroxy-cavernamines-Leu, His, Val, Arg, Trp (**12–16**)		
*Cis*-cavernines (**17**)		
*Trans*-cavernines (**18**)		
Falconensins O (**19)**	*A. falconensis* [35]	Anti-inflammatory (MDA-MB-231 cells line for NF-κB inhibition: 15.7 µM)
Falconensins P (**20**)		Not tested
Falconensins Q (**21**)		Anti-inflammatory (MDA-MB-231 cells line for NF-κB inhibition: 11.9 µM)
Falconensins R (**22**)		Anti-inflammatory (MDA-MB-231 cells line for NF-κB inhibition: 14.6 µM)
Falconensins S = 8-*O*-Acetil-falconensin I (**23**)		Anti-inflammatory (MDA-MB-231 cells line for NF-κB inhibition: 20.1 µM)
Penicitrinol Q (**24**)	*A. terreus* [36]	Antimicrobial (*S. aureus*: 4.3 mg/mL; *B. subtilis*: 6.2 mg/mL)
*Chaetomium*		
Chaephilone C (1R,7S,8R,8aR,9E, 11S,40R,50R) (25)	*C. globosum* TW1–1 [37]	Anti-inflammatory (inhibit NO production: 15.12 µM)
Chaephilone D (**26**)		Anti-inflammatory (inhibit NO production: 20.65 µM)
Chaephilone C * (27)	*C. globosum* [39]	Cytotoxic (HepG-2: 38.6 µM); BST (68.6% of letality at 10 mg/mL)
Cochliodone J (28)	*C. globosum* [40]	Cytotoxic (HeLa: 17.3 µM)
(4′R,5′R,7S,11S)-N-(3,7- dimethyl-2,6- octadienyl)-2-aza- 2-deoxychaetoviridin A (**29)**	*C. globosum* MP4-S01–7 [41]	Antitumor (MGC803 and AGS gastric cells lines: 0.78 and 0.12 µM, induced apoptosis)
4′-epi-N-(3,7-dimethyl-2,6-octadienyl)-2-aza-2- deoxychaetoviridin A (**30**)		Antitumor (MGC803 and AGS gastric cells lines: 0.46 and 0.62 µM, induced apoptosis an altered the cell cycle distribution)
N-(3- methyl-2-butenyl)-2-aza-2-deoxychaetoviridin A (**31**)		Antitumor (MGC803 and AGS gastric cells lines: 2.7 and 6.5 µM)
4′- epi-N-(3-methyl-2-butenyl)-2-aza-2-deoxychaetoviridin A.(**32**)		Antitumor (MGC803 and AGS gastric cells lines: 3.0 and 2.9 µM)
N-(3,7-dimethyl-2,6- octadienyl)-2-aza-2-deoxychaetoviridin E (**33**)		Antitumor (MGC803 and AGS gastric cells lines: 0.72 and 0.12 µM)
N-(3-methyl-2-butenyl)-2-aza-2-deoxychaetoviridin E (**34**)		Antitumor (MGC803 and AGS gastric cells lines: 6.8 and 2.0 µM)
4′,5′-dinor-5′-deoxy-N-(3,7- dimethyl-2,6-octadienyl)-2-aza-2-deoxychaetoviridin A (**35**)		Antitumor (MGC803 and AGS gastric cells lines: 2.2 and 1.2 µM)
4′,5′-dinor-5′- deoxy-N-(3-methyl-2-butenyl)-2-aza-2-deoxychaetoviridin A (**36**)		Antitumor (MGC803 and AGS gastric cells lines: 5. 8 and >10 µM)
Seco-chaetomugilin (**37**)	*C. cupreum* [42]	Anticancer (MCF-7: 75.25% at 50 mg/mL) Increased ROS production: 19.6% at 5 mg/mL
Chaetolactam A (**38)**	*Chaetomium* sp. g1 [44]	Cytotoxic (Not detected)
11-epi-chaetomugilide B (**39**)		Cytotoxic (HL-60: .3.19 µM; A549: 8.37 µM; MCF-7: 4.65 µM; SW480: 4.21 µM; apoptosis induction mediated by caspase 3 in HL-60 cell: 3 µM)
Chaetomugilide D (**40**)		Cytotoxic (HL-60: .15.92 µM; MCF-7: 17.97 µM; SW480: 14.09 µM; apoptosis induction mediated by caspase 3 in HL-60 cell: 15 µM)
Globosumone (**41**)	*C. globosum* [45]	Cytotoxic (Not detected)
*Hypoxylon*		
Hybridorubrin A (**42**)	*H. fragiforme* [47]	Antimicrobial (% biofilm inhibition of *S. aureus*: 81% at 250 mg/mL)
Hybridorubrin B (**43**)		No antimicrobial or cytotoxic activity
Hybridorubrin C (**44**)		Antimicrobial (% biofilm inhibition of *S. aureus*: 82% at 250 mg/mL)
Hybridorubrin D (**45**)		Antimicrobial (% biofilm inhibition of *S. aureus*: 71% at 250 mg/mL)
Fragirubrin F (**46**)		Not tested
Fragirubrin G (**47**)		Not tested
Rutilin C (**48**)		Antimicrobial (% biofilm inhibition of *S. aureus*: 58% at 250 mg/mL)
Rutilin D (**49**)		Not tested
3′-Malonyl-daldinin F (**50**)	*H. fuscum* [48]	Cytotoxic (L929 murine fibroblast: weak; KB 3.1 cervix-cancer cells: weak)
*Monascus*		
Monapilonitrile (**51**)	*M. pilosus* BCRC 38072 [49]	Anti-inflammatory (inhibit NO production: 2.6 µM)
Monapilosine (**52**)		Anti-inflammatory (inhibit NO production: 12.5 µM)
N-Ethanolic monapilosine (**53**)		Anti-inflammatory (inhibit NO production: 27.5 µM); cytotoxic (LPS-induced RAW264.7: cell viability< 65% at 50 µM)
*Muyocopron*		
Muyocopronone A (**54**)	*M. laterale* ECN279 [50]	Antimicrobial (Not detected)
Muyocopronone B (**55**)		Antimicrobial (methicillin-resistant *S. aureus* and vancomycin-resistant *E. faecalis*: MIC at 128 mg/mL)
Lijiquinone 1 (**56**)	*Muyocopron* sp. ** [51]	Antifungal (*C. albicans*: 79 µM; *C*. *albidus*: 141 µM); Cytotoxic (RPMI-8226: 129 µM)
*Penicillium*		
Penicitrinone G (**57**)	*P. citrinum* WK-P9 [52]	Antimicrobial (Not detected)
Dangelone A (**58**)	*P. dangeardii* [53]	Cytotoxic (Inactive: IC > 20 mmol)
Dangelone B (**59**)		Cytotoxic (HepG2: 6.82 mmol; MCF-7: 14.98 mmol)
Dangelone C-G (**60–64**)		Cytotoxic (Inactive: IC > 20 µM)
Dangeloside A and B (**65 and 66**)		Cytotoxic (Inactive: IC > 20 µM)
Didangelone A-H (**67–74**)		Cytotoxic (Inactive: IC > 20 µM)
Tridangelone A-E (**75–79**)		Cytotoxic (Inactive: IC > 20 µM)
Penctrimertone (**80**)	*Penicillium* sp. T2–11 [54]	Antimicrobial (*C. albicans*: 4mg/mL; *B*. *subtilis*: 4mg/mL); cytotoxic (HL-60: 16.77 µM; SMMC-7721: 23.03 µM; A-549: 28.62 µM; MCF-7: 21.53 µM)
*Phomopsis*		
Phomopsone A (**81**)	*Phomopsis* sp. CGMCC No.5416 [55]	Antiviral (Not detected); cytotoxic (Not detected)
Phomopsone B (**82**)		Antiviral (HIV-1: 7.6 µM); cytotoxic (A549: 176.7 µM; MDA-MB-231: 303.0 µM);
Phomopsone C (**83**)		Antiviral (HIV-1: 0.5 µM); cytotoxic (A549: 8.9 µM; MDA-MB-231: 3.2 µM); apoptosis (PANC-1 cancer cells: 28.54% at 17.3 µM
Tersaphilone A-C (**84–86**)	*P. tersa* FS441 [56]	Cytotoxic (Not detected)
Tersaphilone D (**87**)		Cytotoxic (SF-268: 7.5 µM; MCF-7: 7.8 µM; HepG-2: 14.0 µM; A549: 8.3 µM)
Tersaphilone E (**88**)		Cytotoxic (SF-268: 5.6 µM; MCF-7: 5.4 µM; HepG-2: 9.8 µM; A549: 6.7 µM)
*Pleosporales*		
Dipleosporalone A (**89**)	*Pleosporales* sp. *CF09-1* [57]	Cytotoxic (MDA-MB-231: 1.9 µM; HeLa: 2.5 µM; MGC-803: 1.3 µM; MCF-7: 2.1 µM; A549: 1.0 µM)
Dipleosporalone B (**90**)		Cytotoxic (MDA-MB-231: 3.8 µM; HeLa: 3.0 µM; MGC-803: 2.0 µM; MCF-7: >10 µM; A549: 3.5 µM)
*Talaromyces*		
*Trans*-PP-O (**91**) Atrosins S (**92**), D (**93**), E (**94**), H (**95**), L (**96**), M (**97**), Q (**98**) and T (**99**)	*T. atroroseus* [32]	Not tested
Talaralbol A (**100**)	*T. albobiverticillius* [58]	Anti-inflammatory (LPS-induced NO production in RAW264.7 cell: 10.0 µM); 31.0% of inhibitory rate)
Talaralbol B (**101**)		Not detected

* isolated as endophytic of *Polygonatum sibiricum*; ** closest relative being *Muyocopron atromaculans* (MUCL 34983); SF-268 (human glioblastoma carcinoma), MCF-7 (breast cancer), HepG-2 (liver cancer), HeLa (human cervix carcinoma), and A549 (lung cancer), BST = Brine Shrimp test.

### 3.3. Recent Insights in the Biosynthesis of Azaphilones 

The biosynthesis of azaphilones has been reviewed by Pavesi et al. [65] and was also considered in the two latest reviews [31]. Five biosynthetic pathways were exhaustively discussed, which highlighted the comprehensive study of *Monascus* and *Aspergillus* pathways [65]. Furthermore, a thorough study performed about the precise role of ammonium nitrate in the production of *Monascus* pigments showed that some biosynthetic pathways can present changes due to the regulation and expression of several key genes involved [66]. The expression of the gene mppG (MrPigF), responsible for orange pigments, was significantly downregulated with ammonium nitrate addition, and an improvement in yellow pigment production was followed by an upregulated *mppE* expression. Additionally, ammonium nitrate increased the NH_3_ content in the fermentation broth resulting in the increased red pigments yield [66].

Dimeric azaphilones have been described in *the Chaetonium* genus, and the fungal laccase-like multi-copper oxidase gene encoded by CcdJ (CHGG_10025) is believed to dimerize the cochliodones [65]. Cochliodone J (**28**), a new dimeric azaphilone containing a spirotetrahydropyran moiety, was reported, but the mechanism of the spiro ring formation still remains to be determined [40]. Moreover, the unusual fusion between an eight-membered lactam and a six-membered lactone, presented in the structure of chaetolactama A (**38**), has not been investigated yet.

The biosynthetic gene cluster responsible for the sequential and convergent production of azaphilones in *Chaetonium* sp. might count with a hidden gene allegedly responsible for the epimerization of the 7-OH group in chaetoviridin E as well as the oxidation/epoxidation leading to OH groups in C-8a and C-1 positions, followed by methylation of the latter, as in (**41**) [45]. Based on studies with *Monascus*, *Aspergillus*, and *Talaromyces,* two biosynthetic gene clusters were postulated to drive the diverse azaphilones in *H. fragiforme*. However, the biosynthetic dimerizations which led to the compounds (**42**)–(**49**) demand more investigations. This represents a challenge because *Hypoxylaceae* azaphilones are exclusively formed during stromata development, which cannot be induced under laboratory conditions [46]. A reasonable proposal consists on a spontaneous aldol condensation responsible for the heterodimerization of different azaphilones derivatives [46].

The biosynthesis of three different azaphilone skeletons was reported for *P. tersa* FS441. The tersaphilone B (**85**) showed the unique 6/6-6 carbon skeleton with a cleaved tetrahydrofuranyl ring, and the diastereomers tersaphilones D and E (**87–88**) displayed a unique five-membered furan ring open and an epoxide ring in C-8a and C-1 positions [56]. A remarkably biosynthetic proposal was provided to penctrimertone (**80**), which presented a 6/6/6/6 tetracyclic ring system with an unusual aldehyde group in one of the rings [54]. It is supposed to be a citrinin dimer furnished by a citrinin monomer that suffered hydration, oxidation, and reduction affording an orthoquinone methide susceptible to an unusual intermolecular hetero-Diels-Alder reaction with another citrinin molecule [57].

Another interesting observation is the presence of a six-membered ring at the C-3 position of the azaphilones core reported in the *Muyocopron* genus, which is present in less than 10% of the hundreds of azaphilones isolated to date. Regarding the compounds **(54–56),** the gene cluster *lij*. was proposed to control a convergent biosynthetic pathway. The LijE would be responsible for the formation of the aromatic ring with a carbon chain attached to the cyclohexanone ring. Reduction of the acyl ester followed by cyclization and dehydration afforded the azaphilone core. This core would be attached by the C-7 OH group to the acyl derivative formed by previous condensation of acetyl-CoA/malonyl-CoA and C-methylation controlled by the LijA gene. The compounds (**54–55**) also presented a 2,4-dimethyl-3-hydroxyhexanoate moiety that was reported in only eight compounds in this genus. The cyclohexanone ring and 2,4-dimethyl-3-hydroxyhexanoate moiety might be biomarkers of the Dothideomycetes class and constitute a noteworthy point to be more investigated [50].

## 4. Processing and Innovations in Azaphilones Production

Over the last decade, many studies have focused attention on optimizing production of pigments and growth of different fungal species. Many variables that affect the production, as fermentation process (submerged fermentation, solid-state fermentation, larger scale), culture media composition (carbon and nitrogen source, C/N ratio, co-factors, surfactants, tricarboxylic acid intermediates), inoculum type and age (spores and mycelium), temperature, pH, oxygen level and agitation; light, humidity, pigment recovery, extraction, and isolation have been critically discussed by recent reviews [11,31,67,68,69]. Some related aspects of production, processing and innovations in azaphilones production published in 2020 and up to March 2021 are highlighted below.

### 4.1. Overcoming Mycotoxin Issues

**Figure 10:**
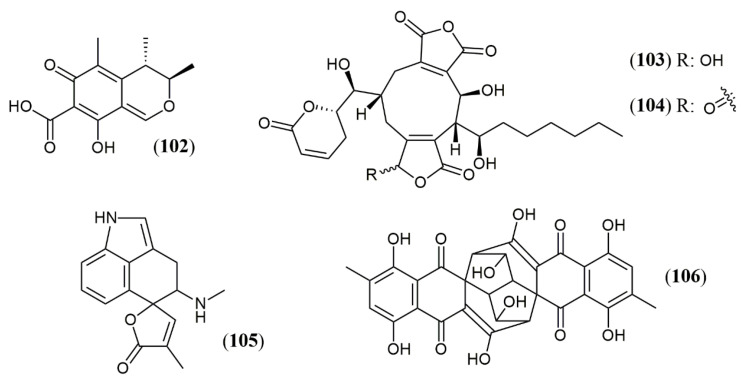
Chemical structures of mycotoxins citrinin (**102**), rubratoxins A (**103**) and B (**104**), rugulovasins (**105**) and luteoskyrin (**106**) [74].

The consensual approval of color additives for food industry by international regulatory bodies is of great importance for commercial transactions, so that in-house products can be exported to other markets without alterations to remove or replace pigments regularized only in the exporting country. US and EU are good examples. Sixteen color additives allowed in the EU are not accepted by US regulatory agency, while four color additives allowed in the US are not permitted in the EU [70]. The ancient knowledge about *Monascus* pigments and utilization of *Monascus* by Asian people for hundreds of years has motivated the search for beneficial and healthy metabolites of *Monascus* azaphilones. Despite the isolation of many *Monascus* metabolites, these pigments were not approved by regulatory agencies in the US and UE so far, due to concerns over co-production of the hepatonephrotoxic mycotoxin citrinin (**102**, Figure 10). Co-production of azaphilones and citrinin is a major issue on this point and optimization of azaphilones production on industrial scale must assure no production of toxic metabolites [71]. For this purpose, genetic techniques have been used, such as depletion of *ctnE* gen, responsible for the production of citrinin (**102**), successfully performed in *Monascus aurantiacus* Li AS3.4384 [72]. The medicinal properties reported for azaphilones are a catalyst in the search for fermentative processes suitable for the production of these pigments from safe biosynthetic routes, obtained by deletion of citrinin gene.

*M. purpureus* has also been studied with the aim of inhibiting citrinin (**102**) production without negative change in pigments biosynthesis. Hong et al. [71] used transcriptome sequencing to explore citrinin gene expression in experiments comparing the effect of inorganic (ammonium chloride and ammonium nitrate) with organic nitrogen (peptone group) sources in *M. purpureus* M3103 metabolism. It was found that biosynthesis of amino acids was up-regulated by ammonium chloride and ammonium nitrate, enhancing the producing of biosynthetic precursors of pigments while essential genes and transcription factors involved in the biosynthesis pathway of citrinin (**102**) were down-regulated by these inorganic nitrogen sources. Therefore, inorganic nitrogen proved to be more favorable for the biosynthesis of citrinin-free pigments (especially orange and red pigments) by *M. purpureus* M3103.

Industry Research and Development Institute in Taiwan is dedicated to investigating new ways to obtain azaphilone pigments using genetic manipulation and optimization of a fermentative process, aiming to avoid the production of citrinin (**102**) (Figure 10). They successfully developed some citrinin-free *Monascus* strains, including the strain *M. pilosus* BCRC 38072, previously mentioned for its production of azaphilones **51–53** [49].

Other mycotoxins are also of concern. *Talaromyces* genus have species reported to produce both, red colorants and mycotoxins (*T. atroroseus* [32], *Talaromyces purpureogenus* [73] and *T. albobiverticillius* [58]) while other species of this genus are not reported to produce known mycotoxins [11,74,75]. Mycotoxins reported from *T. purpureogenus* are rubratoxins A (**103**) and B (**104**), rugulovasins (**105**) and luteoskyrin (**106**), (Figure 10) therefore limiting the use of this species for biotechnological production of food pigments [73]. *T. purpureogenus* CFRM0 produces higher yield of pigments in Potato Dextrose Agar (PDA) and Charcoal Yeast Extract (CYE) rather than in Malt Extract Agar (MEA) and Yeast Extract with Supplements (YES) media (30 °C, 3–4 days), although the growth rate was similar in all conditions [73]. The pigments produced by *T. purpureogenus* CFRM0 were not toxic to female Wistar rats. No alterations related to toxicity were found, including no biochemical, hematological and histological modifications, indicating the safety of this pigment even when administrated in successive days [73].

### 4.2. Color-Directed Production of Pigments

**Figure 11:**
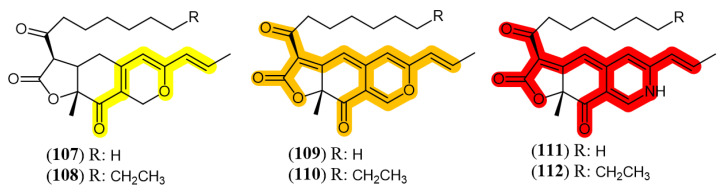
*Monascus* pigments and their chromophores (highlighted in color). Yellow: **107**: monascin; **108**: ankaflavin; Orange: **109**: rubropunctatin; **110**: monascorubrin; Red: **111**: rubropunctamine; **112**: monascorubramine [79].

Fungi from *Monascus* genus are the oldest source of azaphilone pigments and this genus is still considered as one of the most proliferous sources of pigments nowadays [76]. Azaphilones produced by *Monascus* species are usually refered as MonAzPs (*Monascus* azaphilone pigments) and are incorporated in many food products as a natural colorant in China, where MonAzPs exceed 20 thousand tons per year. It is estimated that the number of consumers that eat food containing MonAzPs daily is over one billion people [77]. *Monascus* pigments have predominantly three colors, yellow (monascin (**107**) and ankaflavin (**108**)), orange (rubropunctatin (**109**) and monascorubrin (**110**)) and red (rubropunctamine (**111**) and monascorubramine (**112**)) [78]. The structures of the mentioned substances and their chromophores, the part of the molecule responsible for their color, are shown in Figure 11. Several works focus *M. purpureus* metabolism [66,79,80]. Literature is also rich in reports presenting conditions to drive the metabolism of other fungal species to biosynthesize or to improve the production of pigments.

**Figure 12:**
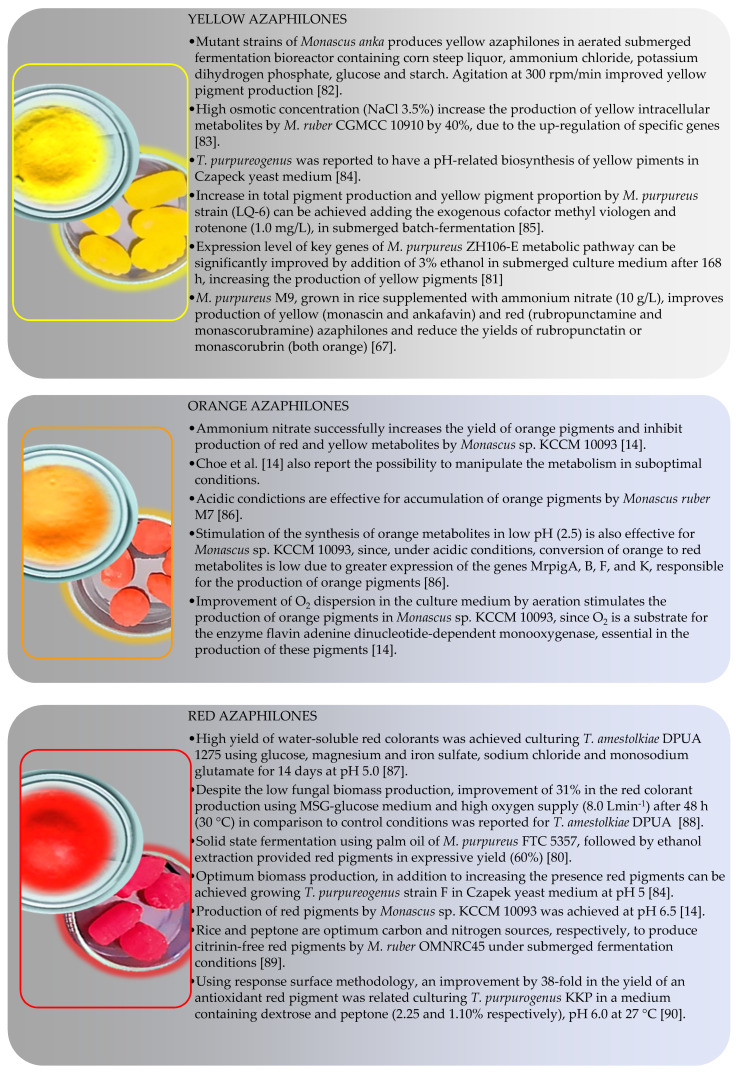
Conditions reported for color-directed production of yellow, orange and red azaphilones [14,67,80,81,82,83,84,85,86,87,88,89,90].

Color-directed production of pigments is advantageous as this approach would eliminate purification steps slowing down the processing by adding a separation step, to purify or concentrate pigments of the desired color. Therefore, a big challenge in pigments production is to obtain pure extracts, containing fewer substances and, preferably, with only one color [19]. Figure 12 presents some fungal species and associated fermentative parameters that resulted in the production of yellow [66,80,81,82,83,84], orange [14,85] or red [14,79,85,86,87,88,89] pigments. However, in most of the works, yellow, orange and red azaphilones are produced simultaneously (cocktail pigments phenomenon) in different proportions.

Regarding *Monascus* species, *M. ruber* CCT 3802 has been studied in terms of colony morphology and biomass production during pigments production utilizing cheese whey as substrate [90]. Strain *M. ruber* M7 showed different response to the addition of acetic acid, sodium acetate and ammonium acetate to PDA culture medium. The original big orange fleecy colony morphology turned into small compact reddish or tightly-packed orange colony upon increase of acetic acid or acetate. Pigment production, in turn, was enhanced by addition of acetate to the culture medium [91]. Yang et al. [16] reported that the expression of key genes for *Monascus* pigment biosynthesis was significantly up regulated in the presence of sodium nitrate. Increase in total pigment production and yellow pigment proportion was reported for a *M. purpureus* strain (LQ-6), after adding exogenous cofactor methyl viologen and rotenone (1.0 mg/L) to the submerged batch-fermentation [84].

The color of pigments produced by *Talaromyces amestolkiae* DPUA 1275 was shown to be pH-dependent. Low pH (2.59 and 3) directed to small production of yellow pigments while red ones were not detected [86]. On a further study, *T. amestolkiae* DPUA 1275 was grown in MSG-glucose medium supplemented with three individual complex nitrogen sources (yeast extract, meat extract and meat peptone), six individual amino acids (glutamic acid, threonine, tyrosine, glycine, cysteine and tryptophan), and two vitamins (biotin and thiamine) [92]. Complex nitrogen and amino acid supplementation did not favor red pigments production but small improvement (1.3 times) was detected after thiamine supplementation.

On the other side, the production of yellow and orange colorants was increased adding yeast extract as nitrogen source in the medium in pH above 5.0. In this condition, conidiation and biomass production were enhanced. The higher yield of colorants in the monosodium glutamic acid (MSG) glucose medium was attributed to the metabolic stress caused by poor nutrition provided by this medium [92]. The production process was scaled-up to a 4 L stirred-tank bioreactor. In another study, the same group [87] evaluated the effect of pH and agitation (100 to 600 rpm) in the improvement of pigments production. They reported near 4-fold increase in orange and red pigments production at 500 rpm, under the pH-shift strategy from 4.5 to 8.0, after 96 h of cultivation at 2.0 vvm at 30 °C. Moreover, the aforementionated work also demonstrated the possibility of using *T. amestolkiae* colorants in the preparation of cassava starch-based biodegradable films for food packaging, resulting in enhancement of protection against butter oxidation, reducing peroxide amount.

### 4.3. Yield Improvement

**Figure 13:**
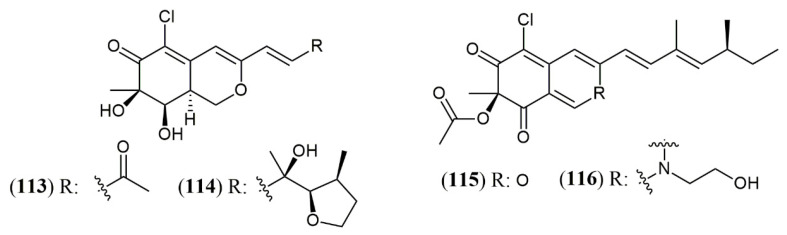
Halogenated azaphilones produced by marine and terrestrial fungi. **113–114**: penicilazaphilones D and E; **115**: sclerotiorin; **116**: N-ethylbenzene-sclerotioramine [95,96].

Yield is another key bottle neck in the way to produce fungal pigments to supply industrial demand. Yield improvement can start early in wet bench step, selecting promising species from under-studied niches. Marine environment has gained prominence in this area in recent decades. In terms of chemical structures, marine metabolites are frequently halogenated in comparison to metabolites biosynthesized by non-marine microorganisms. Halogenated fungal metabolites reach 59.2% of metabolites isolated from marine fungi and, among these metabolites, several halogenated pigments of the azaphyllone class have been reported, as penicilazaphilones D (**113**) and E (**114**) isolated from *Penicillium sclerotiorum* (Figure 13) [38,93]. It is noteworthy that fungal species isolated from marine environment can also be isolated from terrestrial sources, such as *P. sclerotiorum*, that, despite being isolated from soil, was also reported of being capable of producing halogenated derivatives (**115** and **116**) (Figure 13) [94,95].

Enhancement of metabolites yield can be achieved applying stressing conditions during fungal development, aiming at activating unconventional metabolic routes related to the production of substances linked to defense (biotic stress) or adaptation (abiotic stress). This technique is particularly interesting for the production of fungal pigments, since these metabolites are associated with defense against various types of abiotic stress [96]. Abiotic stress is usually caused by altering nutrients (carbon, nitrogen, minerals) and conditions (temperature, length, oxygen supply) in the culture medium, improving pigments production, although independently of directing to a single pigment color. Increase in biomass development is not a must to enhance pigments production, as optimized conditions for development of fungal biomass not necessarily guarantee maximum production of metabolite [82]. In general, in the search for better yields, both, biomass and metabolite yield should increase [97].

The relationship between fungal development and pigments secretion was reported for *T. albobiverticillius* (IBT31667). When cultured on Czapek Yeast Agar (CYA), a malt-free extract, this species produced atrorosins, pigments already reported as metabolites of *T. atroroseus* IBT 11181 [32]. Production of atrorosins by *T. atroroseus* was accomplished on a complex culture medium containing metals solution supplemented with single amino acids as the sole nitrogen source in the range of pH 4–5. In sequence, Tolborg et al. (2019) [97] demonstrated that individual amino acids as the sole nitrogen source led to high biomass production but not necessarily to high amounts of red pigment in *T. atroroseus*. Tolborg’s group also reported that some amino acids can avoid the cocktail pigments phenomenon directing *T. atroroseus* to produce single atrorosins. Corroborating their work, only atrorosin S (**92**) was detected in the fermentation broth when serine was used as the sole nitrogen source. Addition of glutamic acid as a second nitrogen source induced the production of atrorosin E (**94**). Interestingly, only some aminoacids induced atrorosins biosynthesis, since individual supplementation of proline, lysine, asparagine and tryptophan as the sole nitrogen source did not result in atrorosins production by *T. atroroseus* [32]. This strain produced two new azaphilone pigments, talaralbols A and B, along with five known azaphilone metabolites, when subjected to growth under submerged fermentation in malt extract medium (ME) (28 °C, 120 rpm) during 14 days [58].

Pigments production by *T. atroroseus* strain GH2 was studied in two different culture media (pH 5.0, 30 ± 2 °C, 200 rpm, 8 days) [98]. The first one was composed by synthetic Czapek-dox modified medium containing high levels of xylose, with and without nutrients supplementation and the second medium was composed by hydrolyzed corncob, a lignocellulosic waste. *T. atroroseus* GH2 demonstrated a significantly different response to the carbon and nitrogen composition of the culture media, with improved growth and enhanced pigments production in the hydrolyzed corncob medium without any nutrient supplementation. Therefore, *T. atroroseus* was pointed by the authors as a promising pigment-producing microorganism for economically competitive large-scale fermentation at lower cost [98].

Carbon source in the fermentation is a very major parameter to direct fungal metabolism. Parul et al. [83] demonstrated that mannitol is the best carbon source for reproduction and growth of *T. purpureogenus* strain F, but the growth is accompanied by low yield of pigment production, while sucrose causes the opposite effect. The authors correlate this fact to species and strain-specific capacity to produce specific enzymes that will dictate the fungus priorities. Under no stressing conditions and abundant carbon availability, primary metabolism is prioritized and the metabolism will be directed to biomass productions instead of secondary metabolites production [83]. In addition, the rate of carbon source depletion is also important. In large-scale industrial production, the rapid growth of the fungus occurs together with rapid decrease in the carbon source concentration. To avoid decrease of metabolite production rate, the carbon source must be constantly added to the fed-batch fermentation to guarantee a constant concentration of this substrate and, consequently, uninterrupted production of pigments [99]. In the same way, culture medium agitation and aeration ensure better distribution of nutrients and better growth, but at the expense of faster depletion of carbon sources. Therefore, agitation and aeration are factors that must be strictly controlled in industrial production [83].

**Figure 14:**
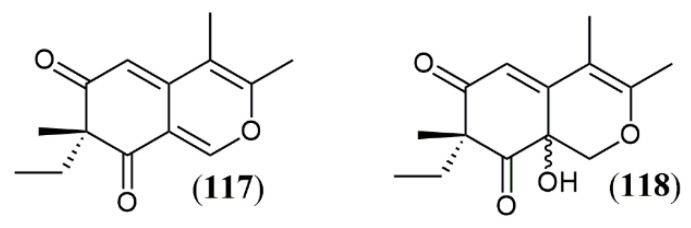
Azaphilones produced by *Plenodomus influorescens* in co-cultivation with *Pyrenochaeta nobilis*: **117**: spiciferinone; **118**: 8a-hydroxy-spiciferinone [102].

Another tool to improve the yield of fungi metabolites is to create stress conditions during fungal development, thar results in activation and/or suppression of gene clusters to allow fungal adaptation and survival. Co-cultivation two fungal species is an example of stressing condition that generates metabolic responses to allow survival in multispecies environment. Oppong-Danquah et al. [100] described a specific co-cultivation gene cluster, when studying the co-culture of pigment producer fungus *Plenodomus influorescens* with *Pyrenochaeta nobilis*, where five polyketides were produced, including the yellow azaphilones spiciferinone (**117**) and 8a-hydroxy-spiciferinone (**118**) (Figure 14). The cultivation of *Trichoderma guizhouense* NJAU 4742 in the presence of *Fusarium oxysporum* cells also resulted in increase in azaphilone production, which was demonstrated experimentally by the increased activity of the gene cluster responsible for pigment production. This fungal response was drove to neutralize the high concentration of H_2_O_2_, produced as a defense mechanism during co-cultivation, since azaphilones are capable of neutralizing free radicals, especially the superoxide anion [101]. The same effect is observed in other oxidative stress conditions related to H_2_O_2_, such as fungal cultivation in the presence of the fungicides amphotericin B, miconazole and ciclopirox. The production of azaphilones increases as a survival mechanism directed to the neutralization of fungicide effects rather than a decrease in antifungal concentration [101].

Cost minimization for industrial production of azaphilones can be reduced by using agro-industrial waste as material for fungal growth, which also helps to solve the problem of pollution associated with the disposal of residues in the environment [19,98]. Liu et al. [102] used rice straw hydrolysate for pigment production by *M. purpureus* M630 but reported that this substrate and does not have the ideal carbon content required by the fungus. Although supplementation may be necessary in some cases, the use of agroindustrial residues has been reported to be economically viable also adding sustainability to the process.

As aforementioned, another approach to achieve yield improvement and consequently increase the viability of industrial production of fungal metabolites is the use of mutant strains and genetic engineering [99]. The current knowledge of the metabolic pathways and secondary metabolism precursors allow to manipulate fungi as “real industrial cell factories” [103] and take advantage of the entire pigment gene cassette to improve pigment yield [104]. In this way, Liu et al. [99] managed to knock-out a cAMP phosphodiesterase gene in *M. purpureus* HJ11, which led to the accumulation of intracellular cAMP causing a stimulating effect in secondary metabolism that resulted in 2.3-fold increase in pigment production.

### 4.4. Extraction Approach

Another phase important in yield improvement consists of the extraction step, which helps in concentration and pre-purification of fungal pigments. Prior to the extraction, it is necessary to take into consideration where the pigments produced are deposited. Classically, the extraction procedure is usually accomplished by liquid-liquid extraction of the broth with medium polarity solvents such as ethyl acetate. This extraction works well to obtain extrolytes, i.e., extracellular metabolites present in the broth or linked to the external surface of fungal biomass. On some occasions, mycelial adhesion is verified, as reported for a water-soluble extracellular yellow pigment produced by a *Monascus* in submerged fermentation. This effect was reversed furnishing sodium and potassium nitrate as nitrogen source to the fungus. Sodium nitrate is suggested to reduce the total amount of extracellular polysaccharides, increase extracellular proteins, and diminish the viscosity of the fermentation broth, rising pigment recovery [16].

Although effective for extraction of metabolites produced in liquid cultures, ethyl acetate is not a choice solvent in terms of toxicity. Non-toxic and easily available ethanol is a better choice for the extraction step, although can only be applied to solid state fermentation, as it is water-miscible and cannot be utilized to extract aqueous liquid media. Ethanol was utilized for pigments extraction in the solid-state fermentation of *M. purpureus* M9 using durian seed as substrate. Extractions were carried out at two temperatures (30 and 60 ^o^C) using a mixture of ethanol and water in different proportions (10:0; 9:1; 8:2; 7:3; 6:4 and 5:5). The most effective conditions for pigments recovery were achieved using the lowest ethanol:water ratios at 30 °C [105].

Occasionally the pigments remain inside the cells requiring disintegration and dissolution of the glucan-chitin complex of the wall cell to be recovered, therefore demanding alternative extraction procedures [106], while cellular lysis is necessary for recovering intracellular metabolites. In this way, *T. amestolkiae* DPUA 1275 was subjected to an alternative extraction procedure to recover red pigments. The procedure was conducted with aqueous solutions of imidazolium salt instead of organic solvents, together with ultrasound-temperature-assisted mechanical cell disruption to enhance the recovery of intracellular *T. amestolkiae* pigments [103].

Cell Pressurized Liquid Extraction technique was utilized to recover pigments produced by mycelial biomass of *Talaromyces* sp. 30570 (CBS 206.89 B) isolated from the coral reef of the Réunion island (France) and cultivated in PDB media containing complex organic nitrogen sources like amino acids and proteins. Eco-friendly solvents were chosen for the extraction (90 °C and 10 MPa) as water, methanol and/or ethanol. At the end, twelve nitrogen-containing azaphilone red pigments were identified while known mycotoxins were not produced [13]. Two-phase aqueous extraction [107] was successfully tested for the extraction of pigments from *T. albobiverticillius*. These organic solvents free techniques guarantee good extraction yields without structural damage in the extracted compounds. Cell disruption methods for improved extraction of pigments from microorganisms were recently reviewed [108].

For pigments production, submerged fermentation is preferable, as it produces better yields, has lower risk of contamination and is easier to monitor when compared to cultivation in solid medium [109]. In addition, using submerged fermentation, it is possible to separate intracellular and extracellular pigments, soluble in the culture medium [102]. However, it is known that not all species of pigment-producing fungi have the ability to diffuse these pigments into the culture medium [73]. Among the techniques to increase the production of extracellular pigments, the design of mutant strains of *M. purpureus* [102], the addition of glycerol to the cultivation medium of *M. pilosus* MS-1 [110] and the establishment of a hyperosmotic environment to *M. ruber* CGMCC 10910 [82] were successfully utilized. The last two methods are related to the regulation of metabolism and gene expression caused by environmental stress.

## 5. Potential Applications of Azaphilones outside Food Sector

As in the food industry, azo dyes represent the most widely used chemical class of dyes in textiles production, an industrial sector that requires high amounts of stable colorants/pigments [111]. Textiles dying quality is also highly important for market competitiveness and consumer identification and public opinion have been driving an increase demand for natural pigments to replace synthetic dyes. In addition, change is necessary to avoid chronic effects in workers exposed to hazardous synthetic dyes during industrial processes. Once present in clothes, aromatic amines can be biotransformed by skin bacteria into aromatic amines, many of which are carcinogenic and can be absorbed by human skin [112]. Non-regulated aromatic amines were detected in a substantial number of colored textiles in a survey done in Switzerland raising questions on genotoxicity, dyes purity, consumer health risks, release of dyestuffs and dermo absorption [113]. Last, but not least, environmental pollution by effluents from textile industry cause multiple environmental harms.

Textile market can absorb some microbial dyes excluded from food applications by regulatory agencies [19]. Toxicity issues and growing preference for natural goods reached clothing sector and many brands are adapting themselves to meet the expectations for sustainable products. This demand increased, especially in millennials and Z-Generation group, as statistics proved to be alarming in global scale in terms of gas emission by textile industry, water contamination and pollution with industrial dyes [114,115]. Modern demands have been raising integrated practices, as well as international networks and partnerships to address sustainability issues and to look for solutions in the textile and clothing industry [115]. This behavior applies to the low-income clothing producing/exporting countries as well as the buyers’ international market. The latter can impose restrictions to imported products containing artificial dyes that either are rejected or avoided by consumers due to the awareness of the unsustainable effluents generated in producing countries.

Cosmetics sector is another market that may incorporate azaphilone compounds in the future. Development of new strategies for on line sales, digital advice, and decentralization of distribution centers helped some cosmetic chains to grow even with the world economic problems associated to the COVID-19 pandemic [116]. The global cosmetics industry was valuated in over USD 380 billion in 2019 and is projected to reach USD 463 billion by 2027 [117]. Several facts contributed to the massive growth of this segment in the last period, such as increase in sale of personal care products, conquering an expressive number of male consumers, increasing number of make-up tutorials in social media and the search for well-being taking into consideration the connection of cosmetics and self-esteem increase [118]. It is also noteworthy that a new type of cosmetics is increasingly growing, named cosmeceuticals. Although regulation of cosmeceuticals was not fully addressed, these products claim biological effects beyond cosmetic utility and many times are referred as cosmetic-pharmaceuticals hybrids.

This expansion in cosmetic market was accompanied by the aforementioned conscientious choice of safe, natural, and “not tested on animals” products [119]. Cosmetics and personal care products are usually directly applied to the skin in a daily basis, many times associated to active ingredients to facilitate fastening or product penetration over the skin. Therefore, allergy and long-term toxicity have also been driving huge efforts for modernization in this area. In this way, long-lasting innovative natural color sources are also an important goal of cosmetics industry. Azaphilone metabolites comprise an important part of the color pallet required by cosmetic industry and their reported biological effects make these compounds also good active components for cosmeceuticals formulations. Anti-inflammatory activity, related for some azaphilone [37,51] is a mechanism associated with anti-aging dermo-cosmetics [120], while antimicrobial activity [46] associated to color pigments can be helpful to extend shelf life of cosmetics.

## 6. Conclusions

The development of new pigments safe and effective to apply in foods, medicines, textile and cosmetic industries is essential and welcome. Natural pigments are a great alternative regarding not being related to toxic, allergic, and pollutant characteristics of the most common synthetic dyes. Fungi azaphilone pigments are recognized as promising candidates of colorants to substitute azo dyes in the food, cosmetics, and textile industrial sectors, as long as safety and production issues are overcome.

Azaphilone research is proliferous and at least 101 new compounds of this class were reported between December 2019 and March 2021 from nine fungal genera (*Aspergillus*, *Chaetomium*, *Hypoxylon*, *Monascus*, *Muycopron*, *Penicillium*, *Phomopsis*, *Pleosporales,* and *Talaromyces*). Some of the new azaphilones exhibit complex chemical structures, and their biosynthesis have been studied to understand nutrients requirements for biomass production and yield improvement. Also, several studies have been conducted to understand down-regulation of citrinin co-production.

Coloring properties and the natural origin are not the only features of azaphilones, since antimicrobial, antioxidant, anti-inflammatory, and other properties related to these molecules have been widely reported. This potential can be explored in food or cosmetic processing to avoid microbial contamination or to furnish functional properties to foods.

This review brought some strategies used to improve fermentation conditions, control pigment production, and issues related to different fungal strains that produce azaphilone pigments, reported in the last two years. Future perspectives include more research that could allow azaphilone dyes to be regularized by the EU, US and other regulatory agencies, so they can be plentiful incorporated in different technological innovative applications.
